# Obstetrics, and Diseases of Women and Children

**Published:** 1860-09

**Authors:** Wm. B. Atkinson

**Affiliations:** Lecturer on Obstetrics, and Diseases of Women and Children


### OBSTETRICS, AND DISEASES OF WOMEN AND CHILDREN.

BY WM. B. ATKINSON, M.D.,

LECTURER ON OBSTETRICS, AND DISEASES OF WOMEN AND CHILDREN.

I.—1. *Statistics of Midwifery.* By H. W. BAILEY, F.R.C.S., London.

The births amounted to 6476; 3290 males, 3186 females. Twins, 53; still-born, 21; triplets, 1; head presentations, 6120; arm presentations, 44; funis, 21. Of these, still-born, 2; breech, 45; face, 40; feet, 180; placenta prævia, 17; fatal hemorrhage, 2; sudden death after labor, no apparent cause, 2; embryotomy, 2; instrumental aid, 113. Deaths of mothers, in all, 13.—(*Obstet. Soc. Lond., Lancet,* Dec. 17, 1859.)

2. *Ibid.* By WM. INGALLS, M.D., Middlesex, Mass.

Cases, 734; births, 746; twins, 12; males, 414; females, 332; abnormal presentations, 39; breech, 10; footling, 5; face to pubes, 14; forehead, 2; occiput posterior, 5; cord and arm, 2; still-born, 6.—(*Boston Med. and Surg. Jour.,* June 7, 1860.)

3. *Ibid.* By A. B. GRANVILLE, M.D., F.R.S., London.

Cases, 12,000; births, 12,478; boys, 6615; girls, 5863; still-born, 413; triplets, 4; instrumental labors, 20.—(*Lancet,* July, 1860.)

4. *Ibid.* By B. L. DODD, M.D., Newark, N. J.

Cases, 100; births, 102; face presentation, 1; breech, 4; average length of cord 26 + inches; still-born, 7; puerperal convulsions, 2.—(*Med. and Surg. Reporter,* July 7, 1860.)

II.—*Management of the Placenta.* By W. NEWMAN, M.D.

After the expulsion of the child, and the division of the cord, the patient still in the usual position, the left hand should be passed over the abdomen to ascertain the condition of the uterus. If contracted, and the placenta extruded from the cavity, it only remains to extract it with the right hand from the vagina. If, however, the uterus be flaccid, and increasing in size, commence and keep up pressure upon that organ until the progressive shrinking of its bulk induces the expulsion of the contained coagula, and the close compression of the placental mass. Then wait some five or ten minutes. After this time, grasp the uterus, and by external pressure cause it to drive the placenta into the vagina, whence it may be withdrawn by seizing an edge with the fingers. Do not trust to the umbilical cord.

The advantages thus secured are the certainty as to the actual state of the uterus; the control obtained over the quantity of blood poured out during the process of detachment of the placenta; the ultimate attainment of complete contraction; the almost complete emptying of the uterine cavity from coagula, etc.; the much increased certainty that concealed or post-partum hemorrhage will not occur; the absence of hour-glass contraction, or undue detention of the placenta.—(*Brit. Med. Jour.*, May 12, 1860.)

III.—*Fœtus carried Twenty-two Months beyond Term.* By James M. Buzzell, M.D., Springfield, Mass.

The patient, aged forty-two, had had several children and miscarriages. In November, 1857, from quickening and other usual signs, she believed herself pregnant. In April, 1858, she was supposed to be in labor; her breasts were so full of milk as to require to be drawn; the pains, however, were not constant or of much force, and soon subsided, although at intervals for two months afterwards, she had pains in her sides. She had menstruated, as was usual with her, two or three times during pregnancy, and afterwards the catamenia appeared at irregular intervals up to the time of her death. After the time of expected confinement, the abdomen gradually lessened in size for six months, when the *tumor*, as it was now supposed to be, was as large as a full-grown fœtus.

In October last she fell down stairs, receiving a severe shock, and afterwards suffering pain and tenderness of the abdomen, with cough and occasional nausea and vomiting. On examination, the os and cervix uteri were entirely closed, the womb forming a solid tumor. On the 19th of February, 1860, she died.

The autopsy revealed extensive adhesion between the fundus uteri and small intestines, and between its side and the sigmoid flexure of the colon. The uterus contained a fœtus in the natural position for delivery, but no trace of a placenta could be found. There was about a pint of thick, yellow fluid in the uterine cavity. An opening in the left side of the womb communicated with the interior of the colon, and the left hand and forearm of the fœtus were passed into the vessel as far as the elbow. Feculent matter had passed into the cavity of the womb.—(*Boston Med. and Surg. Jour.*, June 14, 1860.)

IV.—*Can a Woman remain ignorant of her Pregnancy up to the time of her Delivery?* By R. Scott Orr, M.D., Glasgow.

In the year 1852, I attended a young married woman, aged nineteen, and delivered her of her first child, under the following circumstances: She was supposed to be laboring under inflammation of the bowels. I found no fever, etc.; but, soon after my arrival, she had a violent labor-pain, speedily repeated. Fears having been expressed that she was about to miscarry, her mother assured me that her daughter was not pregnant, having never had any symptom, or felt any motion of a child. An opiate was given to prevent the miscarriage, but the labor went steadily on, and she was soon delivered of a very small infant, apparently still-born; but, by the use of the hot and cold bath alternately, it was resuscitated, and is now living, a fine, healthy boy, eight years old. It could not have been more than six months and a half in utero. The mother assured me she had never had the most remote idea she was pregnant, and the enlargement of the abdomen, which was not very great, she had not paid any regard to. Having been very delicate prior to marriage, her catamenia had been extremely irregular, so that no exact date could be obtained as to the duration of gestation. The patient was in every respect trustworthy, and there was no possible reason for concealment, but, on the contrary, felt proud of her offspring. This case, then, I think, proves that a woman *can* remain in ignorance of her pregnancy up to the *very* time of her delivery.—(*Lancet*, June 30, 1860.)

### V.—*Puerperal Convulsions from Albuminuria, successfully treated with Chloroform.* By CHAS. A. LEE, M.D.

Mrs. T., aged twenty-one, in labor with her first child. Face pale, and with the feet and ankles much swollen. Otherwise apparently healthy, though more or less troubled with headache. Urine loaded with albumen. Fearing convulsions, chloroform was used moderately for three hours prior to delivery, which took place while she was in an unconscious state. In labor eight hours. At intervals, there were twitchings and contractions of the hands, turning up of the eyes, etc. She seemed to be doing well, and being left for an hour and a half, two violent convulsions came on, followed at intervals of an hour by ten more.

The usual means having been employed, choloroform was again resorted to. The patient at first retained her consciousness between the attacks, but finally became perfectly comatose and could not be roused, the convulsions being of an epileptiform character. After commencing the chloroform, but one paroxysm occurred, and that was during the intermission of the anæsthetic. As soon as any of the symptoms appeared, the chloroform was applied, and a few inspirations removed them, and caused entire relaxation. The vital functions were all carried on with regularity. About forty-eight hours after delivery, all threatening symptoms had disappeared, and the urine, on examination, was found to be free from albumen. The patient remained wholly unconscious all this time, and perhaps as long after. The pulse ranged from 150 to 180, sometimes being too feeble to be counted, but gradually came down to 120. Support was given by essence of beef, carb. ammonia, brandy, etc. Twenty-two days from her confinement, she was doing well, sitting up, nursing her infant.—(*Amer. Jour. Med. Sci.*, July, 1860.)

### VI.—*Observations on the Treatment of Puerperal Eclampsia.* By DR. PIRRIE, Belfast.

There is more or less cerebral congestion in these cases, which may be followed in rare cases by effusion, but this is not the *primary* disease. It is a direct consequence of the spasmodic action of muscles of respiration interrupting the oxygenation and due circulation of the blood. He believes true puerperal eclampsia is caused by albuminuria. A condition very analogous, if not identical with anæmia, is produced by the kidneys permitting the exudation of serum, while they retain excrementitious matters, which should have been eliminated, causing the contaminated blood to circulate with difficulty, giving rise to œdema.

Simpson, who was one of the first to demonstrate the intimate connection between albuminuria and puerperal eclampsia, though hesitating to place them in the relation of cause and effect, yet describes them as, perhaps, simultaneous or successive effects of one cause, viz., a pathological state of the blood, to the occurrence of which pregnancy peculiarly disposes the patient.

Dr. P. is more inclined to refer it to congestion of the kidneys, caused by a direct pressure of the gravid uterus. In support of this view, we have the vast preponderance of such attacks in primiparæ, and the rapidity with which the albumen disappears from the urine after delivery. Sir Charles Locock, in the article "Convulsions," in the *Cyclopedia of Practical Medicine*, in assigning mental emotions as excitants of these paroxysms, says: "It has been long remarked, that unmarried women are more liable to be sufferers from convulsions, from the shame and distress under which their children are usually born." When we bear in mind the efforts made by such women to conceal their condition, we may readily conclude that direct physical pressure has more influence in the production of the convulsions in these cases than mental emotions.

The treatment is to relieve the pressure, not on the brain, but on the renal vessels; eliminate noxious matters from the blood; and stimulate the kidneys to a healthy secretion. These rules are applicable, whether the convulsions appear during pregnancy or labor, or after delivery. Thus, we may act freely on the stomach and bowels, reducing the contents of the abdomen, and eliminating noxious matters from the circulation; we may restore healthy urinary secretion by the administration of benzoic and tartaric, or other vegetable acids, as recommended by Braun and Frerichs. Finally, when danger is imminent, evacuate the uterus.

Chloroform and ether are useful adjuvants. In this connection, we must remember the highly important fact discovered by Kussmaul and Tenner, in their experiments, that in animals subjected to etherization, convulsions could not be induced.

While opposed to venesection as a prophylactic and curative agent, as he firmly believes its tendency is to increase the severity of the attack, to debilitate the patient, and delay the recovery, yet he thinks local bleeding, leeches to the temples, or cups to the nape of the neck may be beneficial by moderating the secondary congestion. In lieu of the warm bath, which is very difficult in the majority of cases to employ, he prefers sponging the surface with warm vinegar and water. To recapitulate:—

Convulsions are *not* caused by plethora or congestion of the brain, but by anæmia or defective nutrition.

Depletion aggravates the tendency to convulsions.

All cases of true puerperal eclampsia are associated with and caused by albuminuria, which is most probably caused by renal congestion, the effect of direct pressure.

The indications for the rational treatment are:—

To relieve pressure, the cause of renal congestion.

Eliminate noxious matters from the circulation, and to stimulate the kidneys to healthy secretion.—(*Dublin Quarterly Journal*, May, 1860.)

VII.—*On Ovarian Gestation* By PROF. ARTHUR WILLIGK.

Should the views of Prof. Willigk be confirmed, the current opinions concerning ovarian gestation must undergo considerable change. He agrees with M. Mayer, in doubting the existence of this form of gestation. Prof. Willigk notices a recent case of alleged ovarian gestation, published by M. Alquie, in which he found "ten embryo sacs, with fat, hair, skin, cartilages, and bones," from which he concludes that there was a tenfold impregnation. The improbability of ten ova at once is urged against the admission of this case. In others, he urges that the microscopic examination has been omitted, leaving it a matter of doubt as to the correct description of the matters found. In examining a preparation in the Olmutz Museum, labeled ovarian gestation, he found in the right ovary a cavity the size of a hen's egg, to the lining of which a body was attached by a pedicle supposed to be a fœtus of seven weeks. This proved, under the microscope, to be nothing but connective tissue of the cyst wall, and the fœtus, a solid mass of the same. Another case proved a similar deception.

The professor demands, as a condition for admitting a case as one of ovarian gestation, that the fœtus itself, or undoubted remains of it, shall be found within the fibrous capsule of the ovary; and says no case has yet guaranteed the existence of this variety of extra-uterine gestation. He analyzes several other cases, and disposes of one by Kiwisch, by comparing it to a case in the museum, examined and figured by himself. The right Fallopian tube is closed at its free end, and bears traces of adhesion; the right ovary has a scarred surface, and contains several Graafian follicles and corpora lutea; the two leaves of the broad ligament of the left side inclose a round sac, the anterior wall of which shows a wide irregular rent. This sac contains, besides clots, an embryo partly surrounded by the amnion; the internal surface is nearly covered with fine chorion villi, which at one spot are developed into a placenta. When the posterior fold is traced back, its direct course over the left ovary is seen. This ovary is inseparably united with the abnormal embryo sac, its surface being marked here and there with threads of connective tissue. This case is one of gestation between the folds of the broad ligaments, and he suggests that the one related by Kiwisch is of the same kind.—(*Vierteljahrsch. f. d. Prakt. Heilkunde*, 1859; *Brit. and For. Med.-Chirurg. Rev.*, April, 1860.)

VIII.—*Retained Menses from Imperforate Os Uteri; Operation; Death.* By I. BAKER BROWN, F.R.C.S.

S. L., aged sixteen, had never menstruated. Every month she suffered great pain in the abdomen and back, lasting for a period varying from a few hours to three days, accompanied by all the usual symptoms of menstruation, without any external appearance.

The vagina terminated in a cul-de-sac; the neck of the uterus could be felt in its normal position, but no os was distinguishable. The lips of the os could be obscurely made out as if covered with the lining membrane of the vagina. Per rectum, the uterus could be felt increased in size, with a sense of fluctuation.

It being evident that the case was one of retained menses from congenital closure of the os, it was decided to operate.

Chloroform having been administered, she was placed in the lithotomy position, a pair of sharp-pointed straight scissors were passed along the finger up to the point of obstruction, and gently pressed through it, without opening them; it required very little force to penetrate the membrane. On withdrawing them, a quantity of thick fluid, of the consistence of treacle, but of the color of red-currant jelly, immediately flowed out. By gentle pressure on the abdomen and per rectum, several ounces were discharged. The vagina was syringed out with warm water, a napkin and binder applied, and after she had recovered from the chloroform, one grain of opium was given her, and repeated every six hours. There was not the least hemorrhage. She passed a good night, the discharge having assumed the appearance of natural menstruation. In the evening there was slight sickness, with tenderness of the epigastrium, and hot poultices over the abdomen were ordered, and she took Dover's powder and effervescents. By the next night, a frequent vomiting of dark bile came on, with severe pain at the epigastrium; the bowels not having acted. In spite of all remedies employed, these symptoms continued, and she died on the third day after the operation.

*Autopsy.*—There was no appearance of general peritonitis, but in that portion of the intestines in contact with the uterus, there was evidence of local inflammation, plastic lymph having been thrown out. Every other portion normal.

Cases of this kind are rare, and it is therefore of importance that it should be recorded, as ignorance on this point is apt to do mischief, both to our profession and the public. The feelings of the relations may be severely tried, and the reputation of the surgeon seriously periled, by an expression of opinion as to the non-advisability of surgical interference. The slightest consideration renders it evident that there is no choice but between continually increasing suffering, leading to ultimate death, and the effort to restore the natural channel by surgical means.—(*Lancet*, July, 1860.)

### IX.—*Treatment of Vaginitis by the Glycerole of Tannin.* By M. Démarquay.

*L'Abeille Medicale* gives the following abstracts from a long memoir on glycerin and its applications to surgery and medicine.

"Every one is aware that vaginitis is a stubborn complaint, and extremely difficult to bring under treatment; we, however, believe that we have established a plan by which this disease can be controlled, and whose efficacy is such that during the four years in which we have employed it, we had but one refractory case. This method consists in the application of pledgets of lint steeped in glycerin, treated according to this formula:—

Glycerin, 3 parts.
Tannin, 1–2 parts.

"The tannin is entirely dissolved by the glycerin, and the result is a substance of a beautiful brown color, inclining to yellow, transparent, of a semi-liquid consistence, very readily imbibed by layers of charpie or of cotton, and, after application, not draining out from them, even when held in the vertical position." The application is made thus: the speculum having been introduced, water is freely injected, to remove all the mucus and pus which is spread over the vaginal walls, which are then dried by means of a pledget of lint attached to a long stick. One or more tampons of wadding, well steeped in the tannated glycerin, are then introduced, followed by a dry tampon to retain any drops which may escape from the first. The speculum is withdrawn, and the patient left quiet till the next morning, when all are removed, and again renewed. Four or five such applications complete the cure. As a precautionary measure, for a week longer the patient should make injections two or three times daily of a decoction of walnut leaves, with the addition of sixty grains of alum to the pound. When, in consequence of acute inflammation not permitting the introduction of the speculum, this application cannot be immediately made, the treatment should be commenced by an appropriate diet, baths, and soothing injections.

These tampons are neither painful, nor do they incommode the patient, who can remain in the upright position part of each day. The local effect is coagulation to a certain extent of the mucus-pus which is secreted; change of color in the mucous membrane of the vagina which loses its inflammatory redness; dryness of the walls and tightening of the tissues; disappearance of pain and issue. Other physicians have employed it with like effect. M. Aran has tried simple glycerin in the form of injections into the womb and vagina, for ulcerations of the neck of the uterus, and uterine catarrh. The injections into the womb had to be discontinued, on account of the pain which they produced. It is possible that this was due to impurity of the glycerin.

We have tried this application, but with no encouraging results.—(*Nashville Jour. of Med. and Surg.*, June, 1860.)

### X.—*Fibrous Tumors of the Uterus.*

1. *Observations on Fibrous Tumors of the Uterus.* By M. DÉMARQUAY.

This form of disease is frequent, and presents certain points of interest to the observer—such as their development, symptoms, etc. It is not rare to see a case where the health is not at all affected by the presence of such a tumor; while in other cases very grave symptoms are present, demanding the intervention of the surgeon. But this aid is not always either easy, or even possible, to render. When the os uteri is dilated, or the tumor in the vagina, with a well-formed pedicle, in fact, a true polypus, there are several methods by which to relieve the patient. But when the tumor is contained in the walls of the uterus, the operation is very difficult, if possible; and, moreover, there are often found very frequent and abundant hemorrhages, exhausting and fatiguing by the almost constant uterine contraction and the expulsive pains analogous to those of labor. There is yet a very grave symptom to be considered, the persistent vomiting, which occurs along with the contractions in many cases.

Amussat has occupied much time with this subject, and has published accounts of his operations for the removal of these interstitial tumors. It has also been performed by Blondin, Mourd, Huguier, and other physicians.

The great danger of this operation is, that it cannot always be completed, in which case the life of the female is almost sure to be lost. One of the best surgeons of Paris attempted its performance, was unable to complete it, and the patient died in a few hours.

The early hemorrhage inseparable from these operations may also cause much danger and even death, more especially, when the surgeon is obliged to incise the neck of the uterus to open a passage by which more easily to get at the interstitial tumor; but supposing that the operator may be able to escape all the primary dangers attending it, such as hemorrhage, syncope, peritonitis, still accidents may yet intervene and compromise the life of the patient at a time more or less distant, as may be seen by the following observations of M. Démarquay.

He has four times performed the operation. It is possible to remove the tumor when it does not project into the abdominal cavity, the urgent indication being very precise, but it is not so important in surgery to be able to perform an operation, as to know the results to the females upon whom a like operation has been performed. This is a question for which he is unable to find a solution in the accounts of other operators; they generally record the primary results, but further it is impossible to know. He will demonstrate that his patients who have borne the operation well, have died at a time more or less distant, of the sequelæ.

The first case, aged fifty years, was strong and well formed, but for several years having lost much by the uterus, and suffered greatly in the pelvic region, had become very anæmic; a tumor, twice the size of the fist, was formed in the posterior wall of the uterus.

In operating, the uterus was caught by the forceps and slightly depressed. The neck being dilated, an incision was made on the fibrous body, the membrane of the uterus isolated, and, with the forceps, an attempt was made to draw out the tumor, which failed. Nevertheless it was extracted as by a wire-drawing process.

The operation was tedious, and the patient lost much blood. At first, no bad results ensued, but in a few days she was attacked with metritis, complicated with diarrhœa, and in six weeks she died from these causes.

The second patient, aged forty-five, of a nervous temperament, had become anæmic from the pains and sanguineous discharges. The tumor here was as large as the head of an infant, and in the posterior wall. Her pains were intolerable, with an attempt, as it were by the uterus, to force out the fibrous body. Along with these she had persistent vomitings.

An incision was made on each side, from the neck of the uterus, to enlarge the opening. A similar proceeding was resorted to as in the former case, and no dangerous consequences ensued. No means, however, could check the vomitings, of which she died two months after. The operation had been performed at too late a period.

The third case, aged forty, was analogous to the above. The operation was performed, inflammation followed about the tenth day, which, after much trouble, was subdued; the patient, however, remained in a weak state, and died in twenty months.

The sequelæ of this operation are generally fatal. It is performed when the patient is in an unfavorable condition, and as a last resource. In one case, a lady applied to be delivered of an enormous tumor, which, from the attending circumstances, was refused. Three years after, the discharge and pain had much diminished, she had recovered her vigor and *embonpoint*, and, what is more curious, the tumor had notably diminished. All this was due to the influence of repose, of diet, of the air of the country and the sea.—(*Journal du Progrès*, June, 1860.)

2. *Spontaneous Expulsion of a Fibrous Tumor of the Uterus after Delivery.* By JOSEPH GRIFFITHS SWAYNE, M.D.

A lady, aged thirty-seven, in good health, miscarried at eight months. Six weeks after she suffered with pain and swelling in the left iliac region; at the end of ten days she lost a great deal of blood per vaginam. All these symptoms varied much for some time, being aggravated at each menstrual period. She again became pregnant some two years subsequently, and was delivered of a fine male infant, after a very tedious labor, by the forceps. For a few days all went well, but she was then attacked with fever, and dull, aching pain and tenderness above the left ilium. On examination, the fundus uteri was found to reach nearly as high as the umbilicus; the uterus itself was large and hard, and it was believed that subacute inflammation and softening was going on in the uterine tumor. Dover's powder and fomentations were employed. In the course of a few days, she commenced to pass, per vaginam, pieces of a fleshy substance, which consisted of concentric layers of unstriped fibre, resembling those of the uterus. These continued, along with a sanguineous discharge like the lochia. To promote the expulsion, ergot was given along with the opium and tonics. The uterus gradually diminished in bulk and subsided until, in ten days, it could not be felt above the pubes.

On collecting all the pieces discharged, they formed a mass about the size of a man's fist, and weighed over half a pound. The discharge gradually ceased, and by the end of a month the patient was quite well.

In this case, before pregnancy, the tumor caused pain and hemorrhage at each menstrual period, yet it is surprising how little the process of gestation was interfered with. The tumor, no doubt, was imbedded in the walls and between the fibres of the womb. In all probability, it was much nearer the mucous than the serous surface; thus it was that during the degeneration and absorption of the uterine fibres after delivery, its connection with the uterus, and consequently its vitality, which gave rise to inflammation and ulceration of the uterine fibres between it and the mucous surface, until a sufficient opening having been made, it was cast off piecemeal into the cavity, and thus the natural processes, in this instance, instead of being destructive, as they usually are, were curative, and were attended with the happiest results.—(*Brit. Med. Jour.*, May 12, 1860.)

3. *Seven Cases of Uterine Polyps, with some Remarks upon the Treatment.* By MIDDLETON GOLDSMITH, M.D., Louisville, Ky.

CASE I.—The patient, aged twenty-five, the mother of two children, had suffered one miscarriage. Prior to this she had frequent hemorrhages. A fetid discharge from the vagina had existed for several days, and upon examination, a large polyp was found projecting into the vagina. It was much decomposed, and readily broke down under the pressure of the fingers. She was directed to wash the vagina frequently with water by means of a syringe, under which treatment she recovered quickly and has since borne children. Here it was obvious that the pedicle had been strangulated by the os uteri.

CASE II.—Mrs. W., aged forty, had had several children; subsequently, three miscarriages. Having suffered much from hemorrhage and expulsive pains, an examination revealed an open os with a polyp just within. Ergot was given, and by the next day caused the descent of the tumor, which was then removed by ligation. Recovery complete.

CASE III.—Mrs. ——, aged thirty-five, had borne several children and had several miscarriages; was pale and anæmic from repeated exhausting hemorrhages at irregular intervals. A polyp was diagnosed and ergot given, resulting in her delivery of a large fibrinous polyp by the end of the second day. Recovery complete.

CASE IV.—Mrs. ——, aged thirty, with similar symptoms. The polyp was seized with the forceps and partly torn and crushed. The fetid discharge gradually ceased, but there was no more hemorrhage.

CASE V.—Mrs. ——, aged forty-one; several children, two miscarriages. Since the last miscarriage, the hemorrhage has been so great as to almost destroy her. A small polyp was found and torn off by means of a finger introduced within the os. Recovery slow.

CASE VI.—Miss ——, polyp protruding from the vulva, bi-lobed in appearance, and as large as two turkey-eggs, joined at their sides; the pedicle, about the thickness of the wrist, could be traced a finger's length into the vagina. This she had the power of expelling and withdrawing at will. Ergot was given; and the tumor thus forced down was ligated. In a short time the mass came away, and a perfect recovery followed.

CASE VII.—Ellen, aged twenty-nine; frequent hemorrhage for last two years. Drachm-doses of tincture of ergot were given every three hours, for sixty hours. This caused the tumor to protrude, which was seized and cut off by means of the écraseur. There was no hemorrhage, and she made a good recovery.

At present, surgeons are divided in opinion between excision and the ligature. The danger of the first is hemorrhage; of the second, putrefaction. Another objection to the former is the liability of other parts to be wounded. We must not lose sight of the difference in structure and vascularity between continuous and discontinuous polyps. The first, especially when malignant, produce copious hemorrhage. But the great majority of these tumors are discontinuous and benign. Velpeau favors excision, and, with many others, agrees that but few of these uterine tumors, say one in every hundred, give rise to serious bleeding when excised. He advises that the patient should be placed as for lithotomy, the tumor grasped with long forceps, dragged down, and the pedicle divided with the knife or scissors. If there is no pedicle, divide the tumor a little above its greatest diameter. When it is too large to pass the vulva, it may be sliced away. If within the womb, dilate the os with sponge-tents and give ergot. A pledget of lint, wetted in the tincture of benzoin, placed on the cut surface, and a tampon in the vagina, will check the hemorrhage.—(*Louisville Med. Jour.*, April, 1860.)

XI.—*Description of a Simple Instrument for Inflating the Lungs of Infants Born in an Asphyxiated State.* By DR. WILSON, Glasgow.

This consists of a vulcanized india-rubber ball, about the size of an orange, to which is attached a German-silver tube six inches long, and slightly curved at its free extremity; the tube is closed at the extreme end, but has two eyes, like a catheter, a short distance from the point. The tube is introduced into the larynx, the ball compressed, forcing the contained air through the tube into the lungs. Another hole, much larger, is made in the tube close to its attachment to the ball, through which fresh air is obtained, the thumb covering this, when the ball is compressed, and removed when it dilates.—(*Amer. Med. Times*, July 14, 1860.)

XII.—*Croup and its Treatment with Antiperiodic Doses of Quinine.* By HENRY F. CAMPBELL, M.D., Georgia.

Dr. C. is fully persuaded that croup, as it occurs in the Southern country, has an important relation to malarial fever. At first, it is generally a *neurosis*, manifesting itself in paroxysms, by a series of symptoms, either of a spasmodic or inflammatory character. In cases where spasmodic croup becomes transformed into the membranous variety, the process is interrupted by *remissions*, and even decided intermissions. "It is our object to urge what we consider almost a specific treatment, which is particularly applicable in the early part of the disease, but by no means unimportant even later. We refer to the administration of quinia in efficient doses during the intermissions and remissions characterizing the initial stages of the disease."

The treatment may be divided into that appropriate during the attack, and that during the intermission.

For the first, we require emetics, as ipecac., lobelia; cloths wrung out of cold water; and when these have relieved the paroxysm somewhat, there remaining a hoarseness, and tendency to a return, with great dryness in the laryngeal respiration, turpentine is of great value, given in small and frequently repeated doses.

During the intervals give quinia; keep the patient fully under its influence for two days. This plan will meet both the quotidian and tertian type.—(*Southern Med. and Surg. Jour.*, May, 1860.)

XIII.—*On the Ophthalmia of New-Born Infants.* By DR. FORTOUL, Dijon.

Artificial lactation favors the production of this affection. Two-thirds of Dr. Fortoul's patients died, generally from intestinal pulmonary inflammation, frequently complicated with muguet. One in twenty had consecutive lesions of the ocular membranes, none of whom recovered. Ophthalmia was generally noticed for the first time on the fifth or sixth day after birth. There was frequently a disposition to sclerema and cerebral inflammations. Those comparatively rare cases, from direct contact of syphilitic and gonorrhœal matter, were extremely severe, while the others were of less local danger, but exhibited a great disposition to other affections of the system.

*Treatment.*—Great stress is laid on diet and temperature; avoid local bleeding and purgatives; cleanse the diseased eye by pouring water into it from a sponge; apply nitrate of silver 1 part to 40 of water; seldom use a strong solution, and rarely the solid stick. In syphilitic cases, he uses mercury locally, being fearful of its internal use, from its irritant effect on the intestinal mucous membrane.—(*Amer. Med. Times*, July 14, 1860.)

